# Stages of chronicity in fibromyalgia and pain catastrophising: a cross-sectional study

**DOI:** 10.1186/1471-2474-11-251

**Published:** 2010-10-27

**Authors:** Baltasar Rodero, Benigno Casanueva, Javier García-Campayo, Miquel Roca, Rosa Magallón, Yolanda López del Hoyo

**Affiliations:** 1 Department of Psychology. Centro Rodero, Clínica de Neurociencias, Santander, Spain; 2 Rheumatology Clinic. Clínica de Especialidades, Santander, Spain; 3 Department of Psychiatry. Miguel Servet University Hospital. University of Zaragoza. Instituto Aragonés de Ciencias de la Salud (IACS). Spain; 4 Institut Universitari d'Investigació en Ciencies de la Salut, Hospital Joan March, Universitat de les Illes Balears, Baleares, Spain; 5 Arrabal Health Center. Department of Family Medicine. University of Zaragoza. Instituto Aragonés de Ciencias de la Salud (IACS). Spain; 6 Department of Psychology. University of Zaragoza, Spain. Instituto Aragonés de Ciencias de la Salud (IACS). Spain; 7 REDIAPP "Red de Investigación en Actividades Preventivas y Promoción de la Salud" (Research Network on Preventative Activities and Health Promotion) (RD06/0018/0017

## Abstract

**Background:**

Fibromyalgia (FM) is a prevalent and disabling disorder characterised by widespread pain and other symptoms such as insomnia, fatigue and depression. Catastrophisation is considered to be a key clinical symptom in FM; however, few studies have investigated how contextual factors, such as catastrophisation, might contribute to the duration of the pain. The present research examined the relationship among pain, catastrophic thinking and FM impact, as a function of stage of chronicity.

**Methods:**

In this cross-sectional study, the sample of 328 patients diagnosed with FM was divided into 3 groups based on level of chronicity: Group A (6 months to 2 years, *N *= 46); Group B (2-4 years, *N *= 59); and Group C (more than 4 years, *N *= 223). The three subscales of the Pain Catastrophising Scale (PCS), rumination, magnification, and helplessness, were used as predictors of dysfunction. The Fibromyalgia Impact Questionnaire and the McGill Pain Questionnaire were also administered. A hierarchical regression analysis was performed on the entire sample and, subsequently, for each group to determine the effect of the continuous process variables (castastrophising and pain) on the stages of chronicity.

**Results:**

Total score and PCS subscales were strongly associated with pain and impact in all the stages of chronicity in FM patients (*r *= 0.27-0.73, *p *< 0.05). For Group A, a regression analysis revealed that rumination predicted FM impact beyond the variance accounted for by age and pain. Both magnification and helplessness predicted impact in Group B, and helplessness was a significant predictor of impact in Group C.

**Conclusion:**

These findings provide preliminary evidence that stage of chronicity is an important moderator of psychological vulnerability for FM impact and should be taken into account by tailoring psychological interventions.

## Background

Fibromyalgia (FM) is defined by the American College of Rheumatology (ACR) as chronic (>3 months), widespread pain (axial plus upper and lower segment plus left and right sided pain) and tenderness in at least 11 of 18 tender points [1]. Patients frequently describe sensations of fatigue, sleep disturbances, morning stiffness, symptoms associated with irritable bowel syndrome and affective distress. The prognosis for symptomatic recovery is generally poor, and the estimation for lifetime prevalence is approximately 2% in community samples [2,3]. Most patients report a high degree of impairment in their daily functioning. When compared to patients with other chronic pain conditions, patients with FM report higher levels of pain and functional disability and judge their quality of life as poorer [4]. Moreover, they make extensive use of health services, thus leading to high costs for medical and societal care [5]. The syndrome's pathology is not well understood, and to date, no treatment has proven effective in fully alleviating its symptoms. A number of meta-analyses and reviews have been conducted on pharmacological [6,7] and non-pharmacological [8-10] treatments available for FM.

Psychological treatments seem to have beneficial short-term effects on the key symptoms of FM, but these effects largely disappear over the long term. Most studies to date have addressed the role of psychological factors in the development of chronic pain following acute pain [11,12]. Several psychological constructs have been associated with the prognosis of FM, such as fear-avoidance [13], self-efficacy and personal control [14], pain coping [15] and acceptance [16]. The construct receiving the most attention currently appears to be catastrophising [17]; this attention may be due to the construct's association with negative prognosis. Catastrophising refers to a combination of negative thoughts and expectations regarding pain, and research shows that it is a critically important variable in understanding the experience of pain in rheumatologic disorders and in other chronic pain conditions. Thus, this thought process may be an important target for both psychosocial and pharmacological treatment of pain [18,19].

Indeed, it has been proposed that the development of FM involves an interaction between the experience of pain and catastrophising, but it is unclear when and how this cognitive construct first becomes important [20]. Consequently, there is a need to study the relationship between catastrophising and dysfunction in relation to the development of FM. One approach might be to use cross-sectional comparisons where different stages of chronicity provide a proxy for the development process.

It is also unclear whether the three components of catastrophising (rumination, magnification, and helplessness) contribute equally to the prediction of dysfunction in FM or whether certain components are more predictive than others. Information concerning the relative importance of the components of catastrophising could help treatment providers to tailor interventions to facilitate positive outcomes.

The aim of the present research was to replicate and extend the findings of Sullivan [21] with regard to FM. To this end, the present study examined whether the three components of catastrophising interacted with chronicity (i.e., the length of time the individual had been diagnosed with FM) in predicting the severity of the FM impact (i.e., activity limitations due to pain).

## Method

### Design

This was a multi-centre, cross-sectional study.

### Participants and setting

The study sample consisted of 328 patients from the Pain Clinic, Santander (Spain) and 8 primary care centres in Zaragoza (Spain) during the year 2009. To be included in the study, patients were required to fulfil several inclusion criteria: (1) be between 18 and 65 years old; (2) be able to understand and read Spanish; (3) meet the ACR criteria for primary FM [1]; and (4) have been diagnosed by a Spanish National Health Service rheumatologist. Exclusion criteria included the following: (1) diagnosis of a severe Axis I psychiatric disorder (dementia, schizophrenia, paranoid disorder, or abuse of alcohol and/or drugs) or a severe Axis II disorder that, from the clinician's point of view, might prevent them from following the study protocol; and (2) refusal to participate.

### Measures

#### Demographic and Pain-Related Variables

Each participant was interviewed and asked to provide information about a number of demographic and pain-related variables, including age, work status, duration of FM diagnosis, medications and other medical treatments.

#### Catastrophising

The PCS is a 13-item scale designed to assess the catastrophising cognitions of individuals by asking them to reflect on thoughts or feelings associated with past painful experiences [22]. It can be divided into three subscales: rumination, magnification and helplessness. Each item is scored from 0 (not at all) to 4 (always), and scores range from 0 to 52. The PCS has good temporal stability, internal consistency and validity. The Spanish version of the PCS has been validated by the current study's authors and shows psychometric properties similar to those of the original questionnaire [23].

#### Pain Severity

The McGill Pain Questionnaire (MPQ) was used as a measure of pain severity [24]. It consists primarily of three types of descriptors, sensory words, affective words and evaluative words, which are used by patients to specify subjective pain experience. It also contains an intensity scale and other items to determine the properties of pain experience. For the purposes of the present study, the Pain Rating Index was used. The Pain Rating Index has been recommended as a reliable and valid measure of chronic pain experience. This instrument has a translated and validated Spanish version [25].

#### Fibromyalgia Impact Questionnaire

The Fibromyalgia Impact Questionnaire (FIQ) is a 10-item self-report questionnaire developed to measure the health status of FM patients [26]. The first item focuses on the patient's ability to carry out muscular activities. In the next two items, patients are asked to circle the number of days in the past week they felt good and how often they missed work. Finally, the last seven questions (ability to work, pain, fatigue, morning tiredness, stiffness, anxiety, depression) are measured using a visual analogue scale. This instrument also has a translated and validated Spanish version[27].

### Procedure

The study questionnaires and protocol were approved by the Ethical Committee of the regional health authority, and the patients signed consent forms attesting to their willingness to participate. After consenting to the study, recruited patients were given a battery of questionnaires to complete. These questionnaires included a pain form for demographic variables, the FIQ, the PCS and the MPQ.

Patients were classified into 1 of 3 groups on the basis of the chronicity of their FM disorder. Patients in Group A had been diagnosed less than 2 years ago (*N *= 46), those in Group B had received the diagnosis between 2 and 4 years ago (*N *= 59), and the members of Group C had carried the diagnosis for more than 4 years (*N *= 223).

### Statistical methods

#### Sample size

There are no previous studies assessing the distribution of disease chronicity in patients with FM. The large sample size was chosen to ensure a minimum of 45 patients in each group. Therefore, patients were recruited from both a tertiary care setting (the Pain Clinic, Santander; *N *= 175; 53.3%) and in primary care centres in Zaragoza (*N *= 153; 46.7%).

#### Analysis strategy

In the descriptive analysis of the sample, means and standard deviations were calculated for continuous variables (i.e., age and pain), and percentages were calculated for categorical variables (i.e., gender and treatment setting). Analysis of variance (ANOVA) was used to compare the three chronicity groups. Pearson correlations were used to assess the relationship between pain catastrophising (total score and subscales) and other psychometric variables such as pain (measured with MPQ) and impact of FM (measured with FIQ). A hierarchical regression analysis was performed on the entire sample to determine the effect of the continuous process variables (castastrophising and pain) on FM impact. All analyses were conducted with SPSS 15.

## Results

No patients were rejected because of severe Axis I or Axis II psychiatric disorder. Only two patients (0.006%) refused to participate in the study. The study sample consisted of 328 patients (93.9% women and 6.1% men), aged 18-77 years (mean 49.5 years, SD: 10.6 years), and all of them self-described as from the European ethnic group. On average, the patients had been suffering from FM for 11.3 years (range 6 months - 40 years), and 22.8% had been granted an invalidity pension.

First, the data were summarised and explored. The mean scores on the measures were compared across the stages of chronicity using Pearson Correlation Coefficients. Mean and standard deviations for demographic and dependent variables are found in Table 1. Despite variations across some measures, there were no significant differences between groups for age, pain, the catastrophising subscales or FM impact. Correlations between the catastrophising subscales and pain and FM impact are displayed for each chronicity group in Table 2. In both cases, and in the three groups, catastrophising subscales were significantly correlated with pain and the FM impact.

**Table 1:** Sample characteristics.

	Group A (< 2 years)	Group B (2-4 years)	Group C (> 4 years)
Age	47.0 (9.8)	48.3 (11.0)	50.3 (10.5)
MPQ	38.8 (7.9)	40.2 (8.2)	40.9 (8.3)
FIQ	71.3 (16.4)	70.8 (16.5)	73.6 (16.1)
PCS subscales			
Rumination	10.4 (4.2)	11.1 (4.1)	11.0 (4.0)
Magnification	6.7 (3.7)	6.6 (3.1)	6.6 (3.1)
Helplessness	13.8 (6.2)	15.4 (5.8)	15.5 (5.6)
PCS total	30.9 (14.3)	33.1 (11.9)	33.1 (11.6)

MPQ = McGill Pain Questionnaire

FIQ = Fibromyalgia Impact Questionnaire

PCS = Pain Catastrophising Scale. Values in parentheses are standard deviations.

**Table 2:** Correlations between PCS subscales and pain and impact.

Group	Rumination	Magnification	Helplessness	Total PCS
MPQ (pain)				
				
A (< 2 years)	0.43**	0.44**	0.53**	0.50**
B (2-4 years)	0.27*	0.40**	0.47**	0.42**
C (> 4 years)	0.32**	0.23**	0.36**	0.34**
				
FIQ (impact)				
				
A (< 2 years)	0.76**	0.63**	0.68**	0.73**
B (2-4 years)	0.48**	0.59**	0.58**	0.60**
C (> 4 years)	0.46**	0.38**	0.50**	0.50**

Group A (n = 46), Group B (n = 59), Group C (n = 223). MPQ = McGill Pain Questionnaire; FIQ = Fibromyalgia Impact Questionnaire; PCS = Pain Catastrophising Scale. * p < 0.05, ** p < 0.01.

Following these analyses, the moderational effect of the stage of chronicity was tested, as in the original paper [21], using a hierarchical regression analysis. To test whether stage of chronicity moderated the effect of the catastrophising components (rumination, magnification, and helplessness) on function, the interaction between catastrophising and stage of chronicity was added.

Firstly, the hierarchical regression analysis was performed on the entire sample to test whether any of the interactions between chronicity and the three catastrophising components was more predictive of FM impact than the individual PCS subscales. Age was entered in Step 1 of the analysis but did not contribute significant variance. Pain severity was entered in Step 2 and contributed 24% of variance to the prediction of FM impact, *r*= 0.48, *F *= 95.9, *p *< 0.001. The PCS subscales were entered in Step 3 of the analysis and contributed 8% to the prediction of impact, *r*= 0.56, *F *= 48.0, *p *< 0.001. The three interaction terms were entered in the final step of the analysis, but they did not contribute any additional variance to the prediction of FM impact.

Secondly, the nature of interactions was explored with separate multiple regressions for each level of chronicity. Pain intensity was used as a covariate in order to address whether the catastrophising components contributed to the variance in function beyond the variance accounted for by pain. The results of regression analyses predicting dysfunction in the three chronicity groups are presented in Table 3. For Group A, pain was entered in Step 1 of the analysis and contributed to 30% of the variance in ratings of impact, *F *= 17.91, *p *< 0.001. In Step 2 of the analysis, age was entered but did not contribute significant variance. The three subscales of the PCS were allowed to compete for entry in the next step of the analysis, and only the rumination subscale met criteria in the regression equation, *F *= 38.09, *p *< 0.001. Rumination accounted for 36% of the variance in ratings of FM impact beyond that accounted for by pain.

**Table 3:** Prediction of FM impact for each stage of chronicity

Predicting FM impact in Group A				
Variables		Beta	RÂ²	Pearson r
Step1	Pain	0.55**	0.30	0.55**
Step2	Pain	0.32**	0.66	0.81**
	Rumination	0.61**		

**Predicting FM impact in Group B**				

Variables		Beta	RÂ²	Pearson r
Step1	Pain	0.50**	0.25**	0.50**
Step2	Pain	0.28*	0.42**	0.64**
	Magnification	0.45**		
Step2	Pain	0.30*	0.41**	0.64**
	Helplessness	0.44**		

**Predicting FM impact in Group C**				

Variables		Beta	RÂ²	Pearson r
Step1	Pain	0.46**	0.21**	0.46**
Step2	Pain	0.30**	0.33**	0.58**
	Helplessness	0.39**		

. The impact was measured with the Fibromyalgia Impact Questionnaire, catastrophising with the Pain Catastrophising Scale and pain with the McGill Pain Questionnaire. * p < 0.05, ** p < 0.01.

For Group B, pain was entered in Step 1 and accounted for 25% of the variance in disability ratings, *F *= 19.53, *p *< 0.001. When the three PCS scales were allowed to compete in the next step of the analysis, both magnification (*F *= 18.5, *p *< 0.001) and helplessness (*F *= 18.5, *p *< 0.001) met criteria for entry in the regression equation, and they accounted for roughly the same percentage of the variance (17% and 16%, respectively) in ratings of impact when controlling for pain.

For Group C, pain was also entered in Step 1 and accounted for 21% of variance, *F *= 57.0, *p *< 0.001. The helplessness subscale of the PCS was entered next because it was the only one that met minimum criteria for entry in the regression equation, *F *= 51.17, *p *< 0.001. It accounted for an additional 12% of the variance.

## Discussion

Most reviews of the current literature conclude that psychological interventions in patients with FM are relatively limited [8-10]. To improve treatment outcomes, more evidence is needed from experimental and prospective studies that examine the specific cognitive and behavioural mechanisms responsible for the development and maintenance of chronic pain and disability. Such evidence would help treatment providers to develop interventions tailored to a patient's risk profile.

Although considerable research has been conducted to elucidate the vulnerability factors associated with pain-related disability, the role of vulnerability-relevant contextual factors has not been systematically investigated.

The purpose of the present study was to test the role of FM impact in relation to a thinking process that often accompanies, and appears to worsen, the experience of unremitting pain, namely, catastrophising, as a function of stage of chronicity (i.e. number of years since the FM diagnosis). From the current perspective, the influence of catastrophising on FM impact was considered to be variable and dependent on the context (i.e., duration of diagnosis) in which the catastrophising thoughts occur.

Accordingly, the findings of the present study provide preliminary evidence that the psychological correlates of FM impact change over time. Specifically, regression analyses revealed that stage of chronicity moderated the relationship between the PCS subscales and FM impact. In the group of patients who had been diagnosed with FM for fewer than 2 years, rumination accounted for significant unique variance in FM impact. Magnification and helplessness predicted FM impact over and above the variance accounted for pain severity for patients who had been diagnosed for 2-4 years, and helplessness was the strongest predictor of FM impact in the group of patients diagnosed for more than 4 years.

Additionally, results are concordant with what was expected. Patients who have suffered from FM for less than 2 years are characterised by exaggerated threat appraisals (rumination), which may contribute to the development of an overly cautious or fearful approach to physical activity. In patients who have suffered from FM for more than 4 years, helplessness appraisals may accentuate the impact on function These results are also in line with those of previous studies on chronic pain, in which rumination was the best predictor of severity of disability in patients who had been experiencing pain for approximately 3 years [28], and helplessness was the best predictor of severity in patients who had been experiencing pain for approximately 9 years [29].

The present findings suggest that interventions that consider stage of chronicity as a moderator of vulnerability for impact may yield more positive outcomes than standardised approaches to the management of FM. Cognitive therapies should be more focused on specific assessments (threat or helplessness) depending on the context to optimise treatment outcomes These findings also suggest that there is an additional facet to consider with regard to the relationship between catastrophising and FM impact, one that derives from a contextual view of how thoughts will variably influence behaviour dependent upon history, situation and, of course, stage of chronicity.

Finally, it is notable that catastrophising was a stronger predictor of FM impact than pain itself for the three different stages of chronicity and that catastrophising remained constant over time, despite the fact that FM impact increased. These findings suggest that not only is the *type *of intervention important but also the *timing *of treatment. The findings accentuate the significance of early detection and treatment of patients who are at risk of developing FM and related problems [30]. Intervening early in the course of a pain condition may help prevent maladaptive patterns of pain coping and illness behaviours that are resistant to treatment, and it may have the potential to reduce or prevent the negative impacts of FM that, in turn, will reduce societal and medical costs. It follows that early intervention is far more likely to be effective than interventions administered in the later stages of the condition. Psychological treatments that are initiated shortly after a patient has been diagnosed with FM can help prevent long-term dysfunction and chronicity.

As Sullivan has pointed out [21], the results of our study are limited mainly because our correlational methods cannot unambiguously determine whether catastrophising leads to higher levels of FM impact or vice versa. Given the consistent relationship between catastrophising and FM impact, however, it is clear that there are important contextual processes at work. Experimental, longitudinal, or clinical methods are needed to illuminate these processes. A second limitation of this study lies in the accuracy of the chronicity classifications. One of the main difficulties that FM patients face is failure to receive the FM diagnosis until well after the onset of the disease. It is estimated that there is a 3 year delay in the diagnosis of FM in Spain [31]. Therefore, it is possible that some of the subjects in this research who were classified as being in one of the earlier stages of chronicity had actually been suffering from pain for some time previously. A final limitation concerns the recruiting methods; because half of the subjects were recruited from a specialised clinic, the sample as a whole may not be representative of all patients with FM.

## Conclusion

The findings of this study highlight the important contribution of contextual factors in prolonging the pain condition, and as such, they have clinical implications for the assessment of FM. The study of contextual determinants of psychological vulnerability will play a role in the development of tailored interventions. Recent developments of Contextual Therapies aimed at pain acceptance have shown that such therapies are relevant in the treatment of chronic pain [32,33]. Based on these preliminary but promising findings, we conclude that if patients with FM were to be subdivided consistent with their distinctive contextual cognitive and behavioural patterns, and if interventions were subsequently modified to match these specific risk profiles, the efficacy of psychological treatment programs could be substantially advanced.

## Competing interests

The authors declare that they have no competing interests.

## Authors' contributions

BR, BC and JGC conceived the project. BC and RM performed the clinical diagnosis of fibromyalgia. YLdH, MR and BR collected the data. BR conducted the statistical analysis, and all authors interpreted the results, drafted the manuscript, and read and approved the final manuscript.

## Pre-publication history

The pre-publication history for this paper can be accessed here:

http://www.biomedcentral.com/1471-2474/11/251/prepub
